# Delayed Senescence and Marketability Index Preservation of Blackberry Fruit by Preharvest Application of Chitosan and Salicylic Acid

**DOI:** 10.3389/fpls.2022.796393

**Published:** 2022-03-04

**Authors:** Joel Ernesto Martínez-Camacho, Ramón Gerardo Guevara-González, Enrique Rico-García, Erik Gustavo Tovar-Pérez, Irineo Torres-Pacheco

**Affiliations:** C.A. Biosystems Engineering, Autonomous University of Querétaro, Querétaro, Mexico

**Keywords:** elicitor, shelf-life, chitosan, salicylic acid, *Rubus* sp.

## Abstract

Blackberry fruits are appreciated as a source of nutrients and compounds related to benefit human health. However, they are highly perishable and very susceptible to decay factors. Current methods to improve and maintain blackberry quality are limited in use because of the fruit's fragile physical properties. Regarding these properties, it has been reported that the activities of certain enzymes are linked to senescence and fruit softening processes. This study was aimed to assess the effect of salicylic acid (SA) and chitosan (COS) as preharvest treatments on the physiology related to improving fruit conservation and preserving the marketability index of blackberry fruit. The preharvest treatments were foliar sprayed on blackberry plants at different concentrations. The activities of enzymes superoxide dismutase (SOD), catalase (CAT), phenylalanine ammonia-lyase (PAL), and polygalacturonase (PG) were measured. Total soluble solids (TSS), titratable acidity (TA), TSS/TA ratio, and marketability index (MI) were analyzed after 144 h of storage. The application of 3 mM of SA and 0.25% of COS treatments preserved the MI of blackberries by reducing leakage, red drupelet reversion (RDR), and mycelium presence in the fruit. SA application increased SOD, CAT, and PAL activities. Our results also showed that SA and COS preharvest treatments modified the activity of the cell wall degrading enzyme PG, which might play a role in improving the shelf life and resistance to decay factors of blackberry fruit without any significant effects on physicochemical properties like TSS, TA, and the TSS/TA ratio.

## 1. Introduction

Blackberries (*Rubus* sp.) are aggregate fruits formed by multiple arranged drupelets (Hummer, 2017). In recent decades, blackberries have become highly popular worldwide, mainly due to their distinctive taste, nutritional properties, and high content of compounds that provide beneficial effects to human health (Złotek et al., 2014). However, their thin skin cause leakage and accelerated decay and also make them susceptible to mechanical damage, which can reduce their shelf life (Junqueira-Gonçalves et al., 2016). In addition to their fragile nature, blackberries are susceptible to the occurrence of red drupelets after harvest, this condition is referred to as red drupelet reversion (RDR). This change of color could be attributed to the exposure of pigments after the breaking or collapse of the fruit, which can be aggravated by vibration during transportation and affects their integrity (Clark et al., 2007; Edgley et al., 2019). The main reasons for the rejection of blackberries during commercialization are RDR, leakage of the fruit, and visible decay symptoms. At this moment, cold storage is the main method to extend the blackberry shelf life. Freshly harvested blackberries have been reported to last up to 14 days at 0°C with relative humidity above 90%, although the average shelf life for blackberry fruits is 2–5 days (Perkins-Veazie, 2016). On the other hand, blackberries are non-climacteric fruits, so, harvesting of blackberries close to a fully ripened stage is recommended (Benichou et al., 2018). Numerous studies report improvement in berry's shelf life (Huynh et al., 2019) through diverse treatments such as coatings (Baldwin, 2016), modified packaging (Junqueira-Gonçalves et al., 2016), controlled atmosphere storage (Yahia et al., 2019), sanitation (Horvitz, 2017), and irradiation (Butot et al., 2018). However, most of the research regarding this topic has not been developed under commercial production conditions. These methods require preconditioning of the samples and are not easily available; also some of them are not fully compatible with the current blackberry production model. As the fruits ripen, the alteration in the metabolism leads to changes in the cell wall composition that could result in weakened cell walls and ultimately compromised cell wall integrity (Muro-Villanueva et al., 2019). Moreover, the fruit softening process is related to the activation of certain degrading enzymes including polygalacturonase (PG), responsible for pectin degradation, one of the main components for cellular adhesion (Wakabayashi et al., 2000). The reduction or slowing down in cell wall degradation enzyme activity could enhance physical fruit properties related to decay (Lo'ay, 2017). For example, SA and COS, two commonly used elicitors, have shown positive effects reducing softening of fruits (Lo'ay and El-Boray, 2018; Mannozzi et al., 2018). Additionally, SA and COS have exhibited the potential to prevent fruit decay by activating plant defensive responses (Lucini et al., 2018; Shi et al., 2018; El-Mogy et al., 2019; He et al., 2019; Hidangmayum et al., 2019). These responses can include the phenylpropanoid metabolism activation, which is related to defense activities such as cell wall reinforcement and antimicrobial effects (Taiz and Zeiger, 2006; Li et al., 2012). These enzymatic responses can be activated through the antioxidant systems in plants, which include enzymes like superoxide dismutase (SOD) and catalase (CAT). SOD catalyzes the conversion of superoxide to hydrogen peroxide and molecular oxygen (Saibi and Brini, 2018); moreover, CAT is an indispensable enzyme that directly converts hydrogen peroxide into water and molecular oxygen in reactive oxygen species (ROS) detoxification during stress conditions (Garg and Manchanda, 2009). The high presence of ROS can be detrimental to fruit quality by causing the oxidation of several components of the cell wall affecting its integrity (Halliwell and Gutteridge, 2015). The formation of specialized compounds can also occur *via* enzymatic synthesis through the activation of phenylalanine ammonia-lyase (PAL), which is a key enzyme in the synthesis of phenolic compounds that has been related to defense activities such as cell wall reinforcement, repellent, and antimicrobial activities (Kessmann et al., 1994; Taiz and Zeiger, 2006). Most of the methods reported to date for the conservation of blackberry fruits require postharvest intervention, which can be detrimental to blackberry quality and are limited by its fragile biomechanical properties. Also, preharvest treatments could be more suitable for very fragile fruits such as blackberries. Based on previous reports on other fruits, it was hypothesized that preharvest treatments of SA and COS could improve the blackberry shelf life by modifying the metabolism related to senescence processes. There are no previous reports regarding the effect of preharvest treatments of blackberry plants and fruits with SA and COS, to evaluate the effects on its shelf life, enzymatic activity, and physiochemical properties, which was the purpose of the present study.

## 2. Materials and Methods

### 2.1. Location and Plant Material

The experiment (September 2020) was conducted in a commercial crop field of “Tupi” blackberry plants (4 years old) in compliance with the USDA National Organic Program (NOP), located in Senegal de las Palomas community, Querétaro, Mexico (20°26′10.1″N, 100°05′06.6″W). The climatic conditions were as follows: a sub-humid climate, with a mean annual temperature of 16.5 °C and mean accumulated rainfall of 572 mm. Pruning was carried out according to standard cultural practices for blackberry (late May). Fertilization consisted of organic microbial fertilizers with the addition of organic liquid humic acids (late May and early June, respectively).

### 2.2. Treatments

Salicylic acid (SA, J.T. Baker®, USA) and low molecular weight chitosan (COS, deacetylation degree ≥90%, 50,000–190,000 Da. Alzor® Biotechnologies, Mexico) treatments were prepared at different concentrations (SA 1, SA 2, SA 3 mM, COS 0.25%, COS 0.5% and COS 1%). For our study, plants and fruit were sprayed until a dripping point using a hand-held sprayer between 7:00 and 8:00 am. Plants and fruits sprayed with distilled water were used as controls (9-10 plants per treatment with three replicates). After 5 h of treatment application, fruits were harvested based on commercial criteria. Black and brilliant blackberries with fully developed drupelets located in similar parts of the plant with no evidence of mechanical damage, discoloration, or infection were harvested for the study.

### 2.3. Marketability Index (MI)

**Figure 1:**
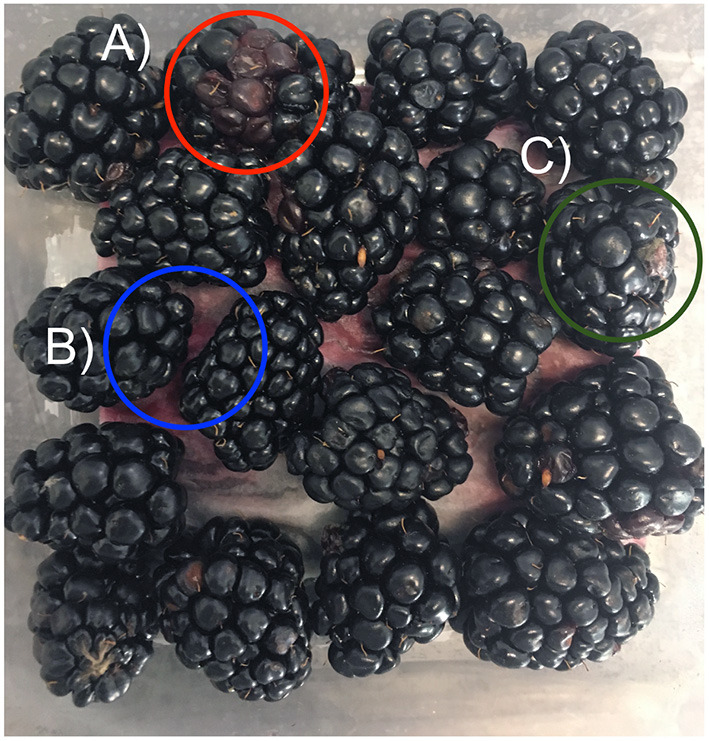
**(A)** Red drupelet reversion (RDR), **(B)** Leakage, and **(C)** Mycelium presence.

The shelf life of the blackberry fruit was estimated by the calculation of an MI. The fruit was collected in commercial 6 oz PET clamshells and put into thermal isolation storage. The samples were kept at 0–1 °C and 90–95% relative humidity for 12 h, then at the ambient temperature of 22–24 °C and 90–95% relative humidity for 132 h. Four clamshells with 12–14 blackberries each were monitored per treatment. Each blackberry was carefully removed from the clamshell container for evaluation. The **RDR**, considered as the occurrence of two or more red drupelets within the same blackberry after harvest, **leakage**, considered as drupelet liquid spillage, and **mycelium presence** considered as presence or absence of visible growth of mycelium in the fruit surface (Figure 1) were registered as percentages every 24 h throughout 144 h. The MI was calculated as follows: MI (%) = 100− [(% RDR + % Leakage + % Mycelium)/3], a minimum value of 85% was considered for a clamshell to be acceptable for commercialization (Clark and Perkins-Veazie, 2011).

Samples from the clamshells were collected after 144 h and stored at −70 °C. Two treatments, COS 0.25% and SA 3 mM, from the MI test were selected for physicochemical properties, enzymatic activity, and total anthocyanin content assays.

### 2.4. Physicochemical Properties

Five blackberry fruits from each clamshell (15 blackberries total per treatment), were selected and homogenized by mixing in a blender. Total Soluble Solids (TSS) were measured as °Brix using a digital refractometer (H196801, Hanna Instruments, USA). TA was measured according to AOAC 942.15 (AOAC, 2000). Titratable acidity was expressed as a percent of citric acid. The TSS/TA ratio was calculated as proposed by Mikulic-Petkovsek et al. (2012). All determinations were assayed in triplicates.

### 2.5. Enzymatic Activity

Polygalacturonase **(EC 3.2.1.15)** activity was determined as viscosity reduction percentage according to Abeles and Biles (1991) and Brummell et al. (2004) with slight modifications. About 500 mg of frozen fruit were grounded with 5 ml of ice-cold 12% polyethylene glycol. Samples were centrifuged at 13,000 g for 10 min at 4 °C. The pellet was incubated for 2 h at 4 °C with 50 mM NaCl/50 mM acetate buffer (pH = 4.4). The samples were centrifuged at 13,000 g for 10 min at 4 °C. The supernatant was recovered as crude enzyme extract. About 1 ml of extract and 4 ml of 2% pectin solution in acetate buffer of 50 mM were mixed. The viscosity was estimated by determining the time required for the solution to pass from the 0.4 to the 0.9 ml mark of a 1 ml pipette and activity was expressed as relative PG activity and registered at five different times at ambient temperature. The lowest relative viscosity at the first measurement was considered as 100%. **SOD (EC 1.15.1.1)**, **CAT (EC 1.11.1.6)**, and **PAL (EC 4.3.1.24)**, activities were determined in a similar way to Beauchamp and Fridovich (1971), Aebi (1984), and Dickerson et al. (1984), respectively. Briefly, 500 mg of sample previously obtained and homogenized were weighted and frozen grounded with 1 to 4 ml of phosphate extraction buffer (50 mM). The sample was centrifuged for 20 min at 10,000 rpm and 4 °C. The supernatant was collected as enzyme extract for SOD, CAT, and PAL determinations. **For SOD activity**, 50 μl of enzyme extract were added to a 2 ml reaction mix containing phosphate buffer (50 mM), nitro blue tetrazolium chloride (NBT 98%, Sigma-Aldrich), methionine (Sigma-Aldrich), riboflavin (Sigma-Aldrich), then the mix was exposed to uniform light for 20 min. Absorbance was read at 560 nm. One SOD unit was defined as the amount of enzyme that inhibits the rate of NBT reduction by 50% under the above assay conditions. **For CAT activity**, 30 μl of enzyme extract were mixed with 100 μl of hydrogen peroxide 100 mM in 1.9 ml phosphate buffer (50 mM). The decrease in absorbance was measured at 240 nm for 1 min. One unit of CAT activity was equal to 1 μ*mol* of H_2_O_2_ degraded per minute. **For PAL activity**, 100 μl of enzyme extract were mixed with 1.15 ml of borate 0.1 M/10 mM L-Phenylalanine (pH = 8.8) buffer. Samples were incubated for 1 h at 40 °C, 0.25 ml of HCl 1 N were added to stop the reaction. Samples were rested for 10 min at ambient temperature then absorbance was measured at 290 nm and compared to a trans-cinnamic acid calibration curve. **Total protein** was determined as Bradford (1976) by Bradford reagent method. Enzyme activity is expressed as U/mg of protein for SOD, CAT, and PAL. COS of 0.25% and SA of 3 mM treatments were selected for enzymatic activity assays. All determinations were assayed by triplicate.

### 2.6. Total Anthocyanin Content (TAC)

Total anthocyanin content was determined in a similar way to Lee et al. (2016) and Paunović et al. (2017) by the pH differential method, with slight modifications. The crude extract was obtained by adding 5 ml of HCl/MetOH of 1% (v/v) to 0.5 g of sample previously grounded and homogenized. The samples were vortexed for 30 s and then sonicated at 45 Hz for 10 min at ambient temperature. The samples were then centrifuged at 4,000 rpm and 20 °C for 5 min, the supernatant was collected as a crude extract. About 1.90 ml of 0.025 M potassium chloride buffer of pH = 1 was added to 0.05 ml of extract, concurrently 1.90 ml of 0.4 M sodium acetate buffer pH = 4.5 was added to another 0.05 ml of extract. Samples were rested for 15 min and the absorbance was measured at 515 nm and 700. Total anthocyanin content was expressed as mg of cyanidin-3-glucoside (C3G) per 100 g of fresh weight.

### 2.7. Statistical Analysis

Data were analyzed by one-way ANOVA. Mean separations were performed by Tukey-Kramer HSD test at α = 0.05. Percentages comparison of RDR, leakage, and decay were performed. A percentage comparison for PG activity was performed using a transformation function. The analyses were performed using IBM® SPSS Statistics v 25 (SPSS Inc., Chicago, IL, USA) and JMP® v 12.1.0 (SAS Institute Inc., North Carolina, USA).

## 3. Results

### 3.1. MI and Individual Decay Factors

In order to estimate the blackberry shelf life, the MI was integrated by three decay factors considered relevant for blackberry commercialization, which was also analyzed individually as shown in Table 1. SA of 3 mM and COS of 0.25% treatments were selected for further discussion since 0.25% of COS presented no statistical difference with the higher COS concentration treatments and SA of 3 mM maintained the MI above the minimum 85% required for the study. The MI of the control group dropped to 68.63% after 144 h, SA of 3 mM and COS of 0.25% significantly preserved the MI showing 92.67 and 93.15%, respectively. The fruit treated with SA of 3 mM and COS of 0.25% showed no incidence of RDR while the control group presented 25% of the fruit affected with RDR. The leakage percentage and mycelium presence in the fruit were also lower with both 3 mM of SA and 0.25% of COS treatments.

**Table 1:** Marketability index (MI) and percentage of individual decay factors present on blackberry fruit after 144 h of storage.

		**Individual decay factors^2^**		
**Treatment**	**Marketability index^1^**	**RDR**	**Leakage**	**Mycelium**
COS 0.25%	93.15% a	0%^*^	9.1%^*^	12.1%^*^
SA 3 mM	92.67% a	0%^*^	11.1%^*^	7.4%^*^
COS 0.5%	91.91% a	0%^*^	12.1%^*^	15.2%
COS 1%	90.87% a	0%^*^	6.1%^*^	18.2%
SA 2 mM	84.87% ab	3.0%^*^	30.3%	12.1%^*^
SA 1 mM	73.96% bc	9.4%	37.5%	31.3%
Control	68.63% c	25%	33.3%	36.1%

1 *t test, α = 0.05. Minimum required 85%. Marketability index was calculated using the per replication data for the individual decay factors. The mean values for RDR, leakage and mycelium presence are presented above*.

2 *Fisher's exact test*.

* *Difference to control at α = 0.05*.

*Different letters indicate significant differences between treatments*.

**Figure 2:**
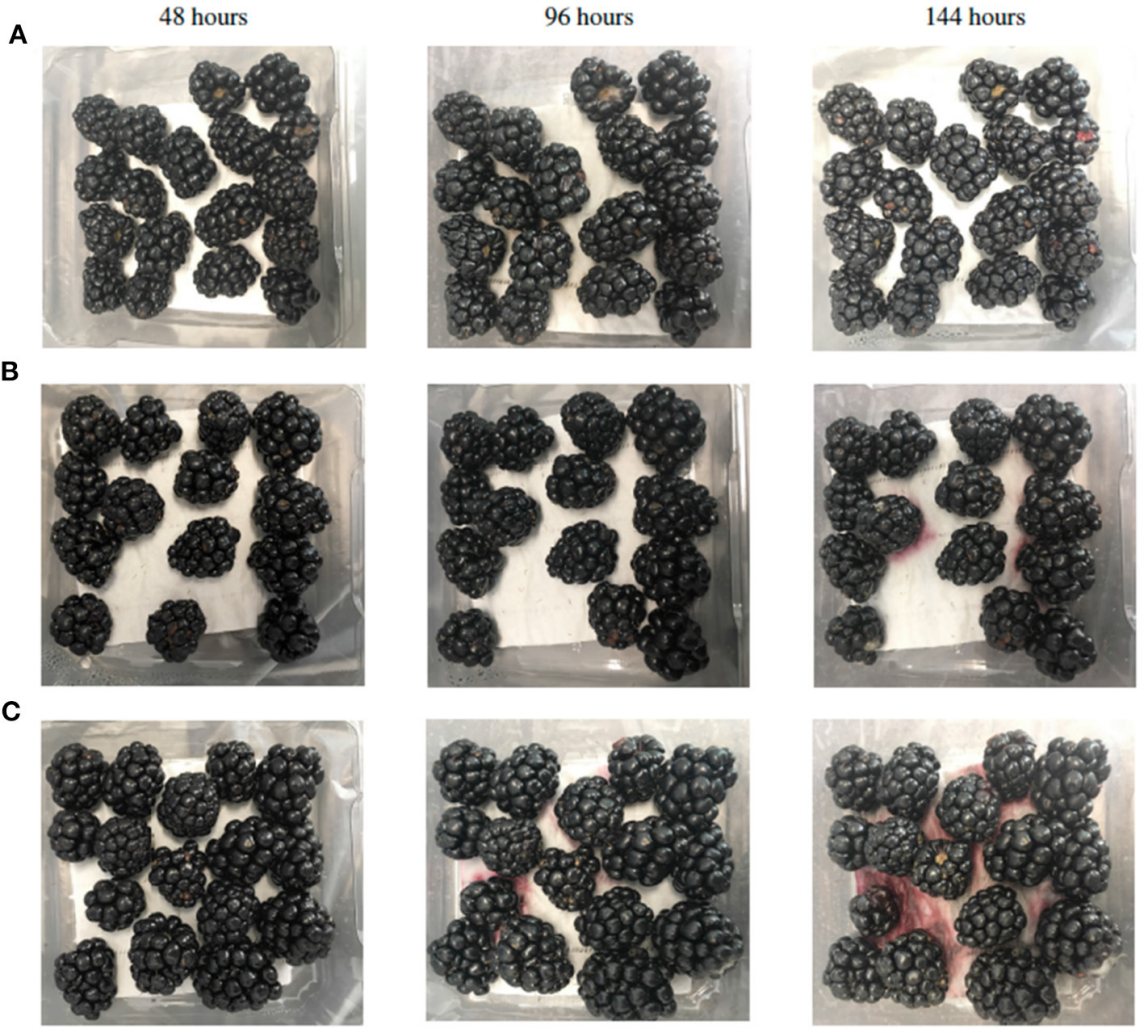
Visual comparison of blackberry fruit at different times of storage, **(A)** COS of 0.25%, **(B)** SA of 3 mM, and **(C)** Control. One representative picture for each treatment is presented.

We documented the changes in the blackberry fruit every 24 h and selected one representative picture for each treatment at 48, 96, and 144 h after storage for a visual comparison. As shown in Figure 2, as the storage time increases, the leakage and mycelium presence in the fruit also tends to increase, especially in the control group (Figure 2C).

### 3.2. Physicochemical Properties

Treatments did not show a significant difference in fruit physicochemical traits evaluated on TSS, TA, and TSS/TA ratio. TSS values were 7.37, 7.47, and 7.46 °Brix. TA had values of 1.90, 1.78, and 1.88%. TSS/TA ratios were 3.88, 4.22, and 4.0 for 3 mM of SA, 0.25% of COS, and control, respectively. In previous reports for blackberries, TSS/TA ratios range from 1.94 up to 14.39 and are highly dependent on the cultivar (Mikulic-Petkovsek et al., 2012; Złotek et al., 2014). Our results showed that the preharvest COS of 0.25% and SA of 3 mM treatments did not affect negatively the organoleptic properties of the blackberry fruit under experimental conditions conducted.

### 3.3. Enzymatic Activity and TAC

There were significant differences in the activity of key enzymes involved in fruit senescence and decay (Table 2). The PG activity, a key enzyme related to fruit softening, was reduced with both COS of 0.25% and SA of 3 mM treatments, showing 73.03 and 80.67%, respectively, compared to 85.33% in the control group. PAL activity, a key enzyme related to defense responses and the phenylpropanoid metabolism, which includes anthocyanin synthesis, increased with both COS of 0.25% and SA of 3 mM treatments. Even though PAL activation was observed with both treatments, only the COS of 0.25% showed a higher anthocyanin content compared to the control. For SOD activity, a reduction was observed with COS of 0.25%, and the activity was significantly higher on SA of 3 mM treated fruit. CAT activity presented no significant difference between the COS of 0.25% treatment and SA of 3 mM treatments, although the CAT activity for the control group was lower than the SA of 3 mM treatment. The 0.25% of COS treatment did not seem to have an effect in the activation of the blackberry enzymatic antioxidant system under the conditions assayed. All together, these results suggest that the 3 mM of SA and 0.25% of COS treatments effectively promoted the activation of the phenylpropanoid mechanism while reducing the activity of cell wall degrading enzymes such as PG.

**Table 2:** Enzymatic activity and total anthocyanin content (TAC) in blackberry fruit after 144 h of storage.

**Enzymatic activity (Unit/mg of protein)^2^**					
**Treatment**	**PGA^1^**	**PAL**	**SOD**	**CAT**	**TAC^3^**
SA 3 mM	80.67 % b	6.27 ± 0.355 a	31.59 ± 0.86 a	76.05 ± 7.43 a	135.96 ± 3.65 ab
COS 0.25%	73.03 % c	3.36 ± 0.015 b	15.00 ± 3.11 c	49.66 ± 4.07 ab	146.37 ± 5.93 a
Control	85.33 % a	1.93 ± 0.021 c	21.00 ± 2.09 b	40.41 ± 16.3 b	133.17 ± 3.40 b

1 *PGA, Relative polygalacturonase activity. HSD Tukey-Kramer test with a transformed value (1+logx), α = 0.05. Data expressed as percentage*.

2 *HSD Tukey-Kramer test, α= 0.05. Data expressed as mean ± SD*.

3 *Total anthocyanin content. HSD Tukey-Kramer test, α= 0.05. Data expressed as mean ± SD*.

*Different letters indicate significant differences between treatments*.

## 4. Discussion

Blackberry fruits are very fragile so the leakage of the fruit content is quite common without the proper handling. The occurrence in RDR is a very specific disorder that happens in blackberries, even though it is still unclear if it affects the fruit properties at a physicochemical level, the change of color exhibited by blackberries affected with RDR is perceived as a sign of bad quality by the consumers. The presence of mycelium is another unmistakable factor for rejection during blackberry commercialization. In general, blackberries tend to be very prone to damage during harvesting and transportation causing the fruit to present some or all of these undesirable conditions. Also, cell wall degradation or mechanical damage can promote pathogen invasion in the fruit skin since most of the pathogens rely on some physical or physiological disorder to invade plant tissue (Kader, 2002). For our study, the presence of leakage and RDR in the control group showed consistency with previously reported observations of higher electrolyte leakage and the presence of RDR (Edgley et al., 2019). The fruits treated with the COS of 0.25% and SA of 3 mM treatments presented lower percentage of leakage compared to control (Figure 2). In this regard, it has been reported that the mechanical damage in cells can promote some pigments to be exposed and change color (Clark et al., 2007). Our results suggested that the reduction in leakage might be associated with increased blackberry resistance to decay factors, specifically pathogen invasion, and RDR; they also showed that SA of 3 mM and COS of 0.25% treatments preserved the fruit for a longer period of time under the assayed conditions. Although different techniques and conditions were used, we found our results consistent with previously reported studies regarding the use of SA and COS on blackberries and similar fruits (Moreno et al., 2015; El-Mogy et al., 2019; Rodŕıguez et al., 2020; Vilaplana et al., 2020).

In general, blackberry fruits soften rapidly once harvested. The application of SA of 3 mM and COS of 0.25% resulted in lower activity of PG in our study (Table 2). The softening process in fruits have been associated with the activation of a range of cell wall-modifying hydrolases which lead to the cell wall disassembly, particularly, PG that has been related to the pectin degradation and cell wall dissolution (Wakabayashi et al., 2000; Brummell et al., 2004; Payasi et al., 2009). The decrease in PG activity observed in our study was similar with previous reports regarding cell wall degradation using SA and COS. Although postharvest treatments were applied, COS and SA could reduce the loss of firmness in fruits by delaying or suppressing the expression and activity of cell wall degradation enzymes like PG (El-Mogy et al., 2019; He et al., 2019; Zhang et al., 2019). Our results suggested that the preharvest COS of 0.25% and SA of 3 mM treatments might reduce or delay the cell wall degrading process and ultimately enhance blackberry shelf life by delaying cell wall decomposition and possibly delaying pathogen invasion.

Anthocyanins are phenolic compounds that have strong antioxidant properties and also provide blackberries with their distinctive color (Paredes-López et al., 2010). The anthocyanin content in our study increased with the COS of 0.25% treatment in comparison to control (Table 2). Our results suggest that COS of 0.25% had a similar effect as reported by Tezotto-Uliana et al. (2014), Barikloo and Ahmadi (2018), and Grande-Tovar et al. (2018) in other fruits. In addition to COS's properties as an activator for the metabolic synthesis of phenolic compounds, it has been reported that COS promotes fruit preservation for longer periods of time (Saavedra et al., 2016; Mannozzi et al., 2018). However, the anthocyanin content with the COS of 0.25% treatment presented no statistical difference in comparison with the SA of 0.3 mM treatment. In our study, the TAC was determined only one time at the end of the storage period; therefore, there is no conclusive information at the moment to determine if COS of 0.25% promoted anthocyanin synthesis, or alternately, it presented a protective effect against anthocyanin degradation in blackberry fruit. It is relevant to mention that the presence of RDR has been related to decreased anthocyanin contents, structural and physicochemical changes in blackberry fruits (Edgley et al., 2019; Kim et al., 2019). This information suggests that the conditions in specific drupelets of the blackberries affected by RDR could lead to the degradation of pigments, including anthocyanins, as well as cell wall components (Clark et al., 2007; Edgley et al., 2019). To our knowledge, there is no consensus to this date between the influence of RDR on anthocyanin content in blackberry fruit, as contrastive results have been reported (Pérez-Pérez et al., 2016; Kim et al., 2019; Flores-Sosa et al., 2021).

It has been reported that ripening fruits have a higher conversion ROS rate due to oxidative processes (Wang et al., 2009). The imbalance between antioxidant enzymes and ROS can cause oxidative stress if there are high radical levels present in the environment or as plant metabolism by-products. The high presence of ROS can be detrimental to fruit quality by causing the oxidation of several components of the cell wall affecting its integrity (Halliwell and Gutteridge, 2015). Some undesirable conditions in fruits like browning, elevated content of hydrogen peroxide, oxidative stress, and fruit ripening are frequently related to SOD and CAT activities (Mondal et al., 2004; Asghari and Aghdam, 2010; Saba and Moradi, 2016). The higher activity in SOD and CAT with the SA of 3 mM treatment might indicate higher protection against oxidative processes within the blackberry fruit. On the other hand, the COS of 0.25% treatment did not seem to have an effect in the activation of the blackberry enzymatic antioxidant system under the conditions assayed.

The phenylpropanoid metabolism which is largely modulated by PAL, has a key role in the resistance against stress factors. The phenolic acids produced *via* PAL have shown antifungal as well as antioxidant activities (Muro-Villanueva et al., 2019). Our results showed an increase in PAL activity, which might have an effect on the synthesis of compounds related to cell reinforcement in blackberry. The activation of the phenylpropanoid pathway is a common defensive response in plants and can promote cell wall reinforcement, repellent, and antimicrobial activities (Haslam, 1988; Taiz and Zeiger, 2006; Muro-Villanueva et al., 2019). It has been observed a significantly positive correlation between anti-fungal activity and total phenolic compound content in plants (Mohamed et al., 2017; Shen and Yang, 2017; Xu et al., 2018). As stated by Muro-Villanueva et al. (2019), down-regulation or block in phenylpropanoid metabolism could lead to accumulation of growth-inhibitory compounds, a reduction in lignin content could result in weak xylem vessel cell walls, vascular collapse, and ultimately compromised cell wall integrity. On the other hand, activation of phenylpropanoid metabolism could result in enhancing the cell wall integrity through the production of additional phenolics (Cocuron et al., 2019). This activation of the PAL enzyme could be ultimately related to the extended shelf life observed in the blackberries treated with COS of 0.25% and SA of 3 mM.

Based on our results, the preharvest application of COS exhibited an overall decrease in the decay incidence in the blackberries, which can be attributed to the inhibition of the PG enzyme and consequently, a reduction in the degradation of cell structures, and the overall antifungal properties of COS (Nia et al., 2021; Wu et al., 2021). Similarly, the impact of preharvest applications of SA on maintaining the MI of the blackberry fruit could be attributed to the reduction in the fruit softening processes and the delay of the ripening by the antagonist effect of SA with senescence-related phytohormones (Garćıa-Pastor et al., 2020b; Gomes et al., 2021; Wang et al., 2021). Even COS and SA showed similar results on the blackberry MI preservation, different mechanisms could be in action for each case. The antimicrobial effects of COS on fruits have been attributed to the polycationic structure by directly interacting and changing the microbial cell wall properties and ultimately inactivating them (Xing et al., 2016; Rajestary et al., 2021). By contrast, the effects of SA on fruits could be related to the effects on the biosynthetic pathways and overall physiological responses in plants (Asghari and Aghdam, 2010; Garćıa-Pastor et al., 2020a).

Blackberry fruit is highly perishable, and it is known to be fragile, which is a limitation to the techniques and methods that can be applied to maintain its quality throughout its commercialization. From our observations, the reduction in RDR incidence and the longer shelf life of the blackberries treated with SA of 3 mM and COS of 0.25% could be attributed mainly to the PAL enzyme activation and the decrease in PG activity. Also, studies are reporting that blackberry handling largely increases the possibility of developing physiological disorders compared with not handled fruit (Edgley et al., 2020). Therefore, to preserve blackberry integrity and shelf life, minimal handling is desired. As mentioned before, most physical and chemical methods reported to date regarding the improvement of blackberry shelf life rely on postharvest interventions. Our results suggested that the application of preharvest treatments focused on modifying blackberry physiology could have positive implications in its shelf life blackberry and make it less prone to decay factors by reducing cell wall degrading enzyme activity, promoting antioxidant enzyme activity, and preventing quality detrimental processes, such as cellular damage, pigment degradation, or oxidative damage.

## 5. Conclusion

In this study, we presented two preharvest treatments for improving blackberry shelf life. Our results suggested that SA of 3 mM and CH of 0.25% preharvest applications could have promising results as a complementary method to improve blackberry shelf life by maintaining fruit physicochemical properties, slowing down the decay processes and activating PAL activity. Preharvest treatments are more suitable for blackberry production by avoiding additional operations after harvesting the fruit. It is important to consider the integration of SA and COS as preharvest treatments as they could represent an economic and technical advantage for blackberry production as a complementary, affordable, and minimal handling method.

## Data Availability Statement

The raw data supporting the conclusions of this article will be made available by the authors, without undue reservation.

## Author Contributions

JM-C: contributed to initial conceptualization for the research, field and lab works, data generation, writing the original draft, and funding acquisition. IT-P: supervised project administration, he also contributed with funding acquisition, field work, and data analysis. RG-G: provided lab facilities, materials, and contributed to reviewing and editing. ER-G: contributed to conceptualization and provided some facilities. ET-P: performed some lab work and provided some materials. All authors contributed to the article and approved the submitted version.

## Funding

This study was partially funded by CONACYT (Ref: 500818).

## Conflict of Interest

The authors declare that the research was conducted in the absence of any commercial or financial relationships that could be construed as a potential conflict of interest.

## Publisher's Note

All claims expressed in this article are solely those of the authors and do not necessarily represent those of their affiliated organizations, or those of the publisher, the editors and the reviewers. Any product that may be evaluated in this article, or claim that may be made by its manufacturer, is not guaranteed or endorsed by the publisher.
